# Dopaminergic control of cognitive flexibility in humans and animals

**DOI:** 10.3389/fnins.2013.00201

**Published:** 2013-11-05

**Authors:** Marianne Klanker, Matthijs Feenstra, Damiaan Denys

**Affiliations:** ^1^Netherlands Institute for Neuroscience, Institute of the Royal Netherlands Academy of Arts and SciencesAmsterdam, Netherlands; ^2^Department of Psychiatry, Academic Medical Center, University of AmsterdamAmsterdam, Netherlands

**Keywords:** dopamine, cognitive flexibility, obsessive-compulsive disorder, reversal learning, set-shifting, task switching

## Abstract

Striatal dopamine (DA) is thought to code for learned associations between cues and reinforcers and to mediate approach behavior toward a reward. Less is known about the contribution of DA to cognitive flexibility—the ability to adapt behavior in response to changes in the environment. Altered reward processing and impairments in cognitive flexibility are observed in psychiatric disorders such as obsessive compulsive disorder (OCD). Patients with this disorder show a disruption of functioning in the frontostriatal circuit and alterations in DA signaling. In this review we summarize findings from animal and human studies that have investigated the involvement of striatal DA in cognitive flexibility. These findings may provide a better understanding of the role of dopaminergic dysfunction in cognitive inflexibility in psychiatric disorders, such as OCD.

## Introduction

In a constantly changing environment behavior has to be adaptive and flexible. Cognitive flexibility is the ability to adapt goal-directed behavior in response to changing situational demands. Cognitive flexibility is one of the cognitive domains that are grouped together as executive functions or executive control (Gilbert and Burgess, 2008). Despite the necessity of cognitive flexibility for everyday functioning there is a substantial variation within the healthy population (Miyake and Friedman, 2012) that can be related to variations in dopamine (DA) related genes in humans (Braver et al., 2010; Barnes et al., 2011) and mice (Laughlin et al., 2011). Specific deficits in the ability to flexibly update behavior are observed in various neurological and psychiatric disorders such as Parkinson's disease, schizophrenia, autism, addiction and obsessive compulsive disorder (OCD) (Cools et al., 2001; Chamberlain et al., 2006; Verdejo-Garcia et al., 2006; Ceaser et al., 2008; Yerys et al., 2009).

Here, we intend to provide an overview of animal and human studies on the relation between cognitive flexibility and DA neurotransmission and relate this to OCD, a psychiatric disease that combines defects in cognitive flexibility and alterations in DA processes.

### Testing cognitive flexibility

The successful adaptation of behavior following changes in the environment encompasses several cognitive processes, such as associative learning, decision making, response selection and inhibition, working memory and attention. Several neuropsychological tests have been constructed to study different types of cognitive flexibility, which may recruit varied cognitive functions and depend on parallel neurobiological substrates. The use and translational applicability of a number of these tasks was discussed by Barch et al. (2009). One set of tasks probes flexibility of choice behavior, where selection of one from two or more options leads to a wanted outcome. For a specific response to be adapted, the behavior has to be acquired first. During discrimination learning, subjects learn to discriminate between a certain rewarded/correct stimulus, strategy or response rule and another one that is not rewarded/correct. When task demands change, the response that has been successful so far no longer yields reward and has to be inhibited, whilst another response/stimulus/strategy has to be chosen, initiated and maintained. This requires extinction of the old association and acquisition of a novel association. Classical reversal learning and intra- and extradimensional attentional set-shifting fall in this category.

#### Reversal learning

With reversal learning, the ability to adapt behavior in response to a reversal of reinforcement contingencies is studied. This requires a shift in valence between stimuli or locations that have been associated with a specific outcome (e.g., a reward) previously. Depending on the operationalization of the reversal task used, this can be a reversal of all sorts of cues, but the choice options remain the same.

#### Attentional set-shifting and strategy shifting

Attentional set-shifting requires adaptation of behavior following changes in the relevance of perceptual categories or dimensions. In an *intradimensional* set-shift, new stimulus exemplars (i.e., novel choice options) are presented but the relevant stimulus dimension does not change between trials. Successful shifting requires maintenance of the current rule (attentional set) and adapting behavior accordingly. In an *extradimensional* set-shift, not only are the stimulus exemplars novel, but the reinforced dimension has also changed. This requires a response shift to a dimension that has previously been irrelevant and bypassing of an acquired attentional bias (Rogers et al., 2000).

In human subjects, the ability to shift cognitive sets is commonly tested with the Wisconsin Card Sorting Test (WCST). The WCST requires matching of a multi-dimensional cue card to one of four reference cards according to a specific stimulus aspect. The attentional set-shifting task has been developed as a non-human primate version of the WCST (Roberts et al., 1988). Because it is a more direct measure of the ability to shift cognitive set and a better measure for frontal lobe impairments (Rogers et al., 2000), it is now often used in human subjects as well.

Both reversal learning and attentional set-shifting paradigms have been developed for humans, non-human primates and rodents. Stimulus dimensions consist of different visual stimulus sets that can be simple or compound in nature (human, non-human primate, rodent) or stimulus sets consisting of multiple sensory dimensions (spatial, odor, touch, visual); rodent bowl digging procedure (Birrell and Brown, 2000; Garner et al., 2006). Discriminations based on stimulus valence have been classified as representing a lower order of abstraction, whereas discriminations based on stimulus components or abstract rules may represent a higher order of abstraction (Wise et al., 1996; Ragozzino, 2007).

Another example of a procedure based on a response rule or strategy and an unannounced switch to a different rule or strategy is response-based versus cue-based responding on a T-maze, often applied in rodents (Packard, 2009).

A general problem with switching responses in these tasks is that several processes occur simultaneously and that incorrect responses may reflect different mechanisms, i.e., resistance to extinction versus learned irrelevance (Maes et al., 2004). Task adaptation (Tait and Brown, 2007) or detailed analysis (e.g., Dias et al., 1996a) lead to more informative outcomes. Three-choice paradigms have been used in non-human primates and may offer superior experimental approaches as they allow testing of more variable conditions and require animals to trace the value of several alternative options, as a change in one option does not automatically imply a change in the other alternative options (Walton et al., 2010).

#### Task switching

Task switching is a paradigm that is mostly, but not exclusively (Stoet and Snyder, 2003; Leenaars et al., 2012) used in human subjects and requires the rapid switching between stimulus-response sets that have been acquired previously (Sohn et al., 2000; Monsell, 2003). Presentation of an external cue indicates which task (stimulus-response set) has to be executed in a given trial. This differs fundamentally from reversal learning and set-shifting procedures, where the presentation of altered contingencies (i.e., “the switch”) is not cued and subjects have to use the change in reinforcing feedback to adapt behavior accordingly.

#### Control over prepotent or automatic responses

Another category incorporates tasks that probe the ability to behave flexibly in conditions that previously allowed automatic or habitual performance. A well-known example is the countermanding or stop-signal task (Logan et al., 1984; Eagle et al., 2008), testing inhibitory control over actions. Another example is the anti-saccade task where a more or less automatic action needs to be suppressed to allow flexible responding (Munoz and Everling, 2004). In the present review we focus on studies using reversal learning, attentional set-shifting (including WCST) and task-switching as these tasks have received most translational interest, have been related to DA function and have been performed in OCD patients.

### Neural circuitry supporting cognitive flexibility

#### Prefrontal cortex

Within the prefrontal cortex (PFC), damage to different prefrontal areas results in dissociable deficits in separate forms of cognitive flexibility. Damage to the orbitofrontal cortex (OFC) is thought to specifically impair reversal learning, but not attentional set-shifting (Dias et al., 1996a; McAlonan and Brown, 2003; Hornak et al., 2004; Boulougouris et al., 2007). Damage to the lateral PFC [or medial PFC in rodents, suggested to be functionally equivalent; (Uylings et al., 2003)] specifically impairs (extradimensional) shifting of attentional sets but not reversal learning (Owen et al., 1991; Dias et al., 1996a, 1997; Birrell and Brown, 2000; Bissonette et al., 2008). However, the proposed unique role of the OFC in reversal learning is under discussion and alternative views have been presented (Schoenbaum et al., 2009). Recent findings suggest that impaired reversal learning in Rhesus monkeys is only observed following aspiration but not excitotoxic OFC lesions (Rudebeck et al., 2013), suggesting that reversal learning does not depend on an intact OFC but instead on intact communication between other prefrontal areas and more caudal structures. While human brain lesions generally involve passing fibers and brain parenchym, many studies in rodents and new world monkeys report deficits after fiber-sparing lesions. The transient character of impairments in these studies may reflect evolution-related differences in neurobiological and/or anatomical substrates of reversal learning (Rudebeck et al., 2013).

#### Striatum

Reciprocal projections from PFC to the striatum and thalamus form parallel frontostriatal loops, suggesting striatal regions also contribute to the regulation of cognitive flexibility (Rogers et al., 2000; Floresco et al., 2006a; Ragozzino, 2007; Clarke et al., 2008; Castane et al., 2010). Combined results from lesion and functional imaging studies suggest that different types of cognitive flexibility are regulated by segregated fronto-striatal circuits: OFC and dorsomedial striatum (human/non-human primate: caudate nucleus; functional equivalent rodent area: dorsomedial striatum) are implicated in reversal learning (Divac, 1971; Dias et al., 1996a; Rogers et al., 2000; McAlonan and Brown, 2003; Bellebaum et al., 2008; Clarke et al., 2008; Castane et al., 2010; Ghahremani et al., 2010). Set- and task switching performance relies on connections between the dorsolateral PFC (or the medial PFC in rodents which is in this task functionally equivalent) and striatum (Owen et al., 1991; Dias et al., 1996a,b; Birrell and Brown, 2000; Sohn et al., 2000; Manes et al., 2002; Ragozzino, 2007; Graham et al., 2009). It should be noted that these circuits are not fully segregated but overlapping. Importantly, these circuits show consistent similarities between primates and rodents (Mailly et al., 2013).

### Dopamine

DA is an important neuromodulator in fronto-striatal circuits. A substantial amount of work has described a role for DA in reward-related learning and motivated behavior. More specifically, burst firing of DA neurons (associated with phasic DA release) may code a quantitative prediction error that serves as a teaching signal to guide behavior and is essential for a range of learning situations (Montague et al., 1996; Schultz et al., 1997; Schultz, 2013; Steinberg et al., 2013). Yet not much is known about the contribution of DA to the adaptation of behavior following changing task demands, such as a reversal of contingencies. A common factor in all tests of cognitive flexibility is the expectation of a reward (or absence of punishment) when a correct response is made. The absence of an expected reward and presence of an unexpected reward following a reversal or shift is the archetypal situation for the occurrence of reward prediction errors coded by DA. Therefore, one would expect that DA is in some way involved in the regulation of cognitive flexibility. However, in the past decade the role of the PFC and its serotonergic innervation in cognitive flexibility received most attention (e.g., Robbins and Arnsten, 2009).

In this review, we summarize findings from animal and human studies that investigated whether DA contributes to the regulation of cognitive flexibility. First, we will describe pharmacological manipulations to the DA system in humans and animals, then DA-related genetics in humans and animals. Next, we report on DA changes and cognitive flexibility in OCD, to investigate whether alterations in DA signaling contribute to cognitive inflexibility in this disorder. Previously, OCD has been proposed to be characterized by a hyperdopaminergic state (Denys et al., 2004b) and similar states in animals have repeatedly been described as leading to OCD-like behaviors (see further). This, combined with the suggestion that impairments in the ability to flexibly adapt behavior may be an endophenotype for OCD (Robbins et al., 2012) drove us to review the evidence for a relation between the two.

## Pharmacological manipulations and imaging studies in human subjects

### DA synthesis

DA synthesis capacity in humans is determined after administration of radio labeled F-DOPA or F-tyrosine and imaging the resulting fluorinated amines using PET. The observed variations in DA synthesis capacity may relate to variations in DA neurotransmission, as a significant negative correlation between synthesis capacity and D_2_- receptor availability was reported (Ito et al., 2011). Decreasing DA synthesis by dietary omission of DA precursors tyrosine and phenylalanine reduces occupation of D_2_ receptors by endogenous DA, suggesting decreased DA transmission (Montgomery et al., 2003). Administration of the tyrosine hydroxylase inhibitor alpha-methyl-paratyrosine also reduces D_2_ occupation by endogenous DA (Verhoeff et al., 2003), but affects noradrenergic signaling as well (Krahn et al., 1999).

The small number of studies using these approaches does not support a general relation between DA synthesis and flexible updating of task information: no correlation was observed between DA synthesis capacity and task performance on the WCST (Vernaleken et al., 2007), and reward- and punishment-based reversal learning was not impaired following DA depletion in males (Robinson et al., 2010). In contrast, catecholamine depletion (affecting both DA *and* NA) impaired performance during probabilistic reversal learning (Hasler et al., 2009).

Other studies suggest that when tasks are used that allow more selective approaches, a differential involvement of DA synthesis is observed. Thus, subjects with high DA synthesis capacity perform worse compared to subjects with low DA synthesis capacity when presented with shifts in object features but not in abstract rules in a task-switching paradigm (Dang et al., 2012). Cools et al. (2009) reported that individuals with high DA synthesis capacity perform better when presentation of an unexpected reward signals reversal compared to reversals that are signaled by presentation of an unexpected punishment, whereas the opposite is observed for individuals with low DA synthesis capacity. Females tend to have a higher DA synthesis capacity (Laakso et al., 2002) and this may explain gender-related differences such as the DA depletion-induced improvement of punishment-based but not reward-based reversal learning in females (Robinson et al., 2010).

In conclusion, DA synthesis is differentially associated with task features in cognitive flexibility and variations in synthesis capacity affect performance only in some task conditions, probably depending on specific DA homeostasis parameters in cortical and striatal areas (cf. Cools and D'Esposito, 2011).

### DA receptor/transporter binding

Using imaging techniques, baseline availability of DA receptors and transporters can be investigated and related to task performance. Receptor availability in resting conditions provides an index of the number of receptors unoccupied by the endogenous transmitter. Subjects with higher availability of DA transporters in the striatum make less perseverative errors in the WCST (Hsieh et al., 2010) but the interpretation of this finding depends on whether the higher availability reflects the density of the DA innervation or a possible substrate-induced adaptation (Chen et al., 2010).

WCST performance has also been linked to differences in DA receptor availability (see Table 1). Decreased striatal D_2_ availability is associated with impaired performance (Volkow et al., 1998), but D_2_/D_3_ receptor binding in the anterior cingulate cortex correlates positively with the number of errors made in the WCST (Lumme et al., 2007).

**Table 1:** **Summary of effects of pharmacological manipulations to the dopamine system on cognitive flexibility in human subjects**.

**Paradigm**	**Manipulation**		**Performance**	**References**
**WCST**				
	Depletion		↓ response times	Nagano-Saito et al., 2008
	Synthesis capacity		=	Vernaleken et al., 2007
	D_2_ agonist		↑	Kimberg et al., 1997
	D_1_/D_2_ agonist		=	Kimberg and D'Esposito, 2003
	DAT availability	Striatum	↑ reduced errors	Hsieh et al., 2010
	D_2_/D_3_ binding	Anterior cingulate		Lumme et al., 2007
	D_1_ binding	Dorsolateral PFC		Takahashi et al., 2008
**REVERSAL LEARNING**				
	DA Depletion		↑ punishment based reversal	Robinson et al., 2010
	Catecholamine depletion		↓ probabilistic reversal	Hasler et al., 2009
	Synthesis capacity	High	↑ reward based reversal	Cools et al., 2009
		Low	↑punishment based reversal	
	D_2_ agonist	High DA	↓ more errors, longer RT	Mehta et al., 2001
		synthesis Low	↓ reward based reversal	Cools et al., 2009
		DA synthesis	↓ reward based reversal	
	D_2_ antagonist		↑ reward based reversal	van der Schaaf et al., 2012
**TASK SWITCHING**				
	Synthesis capacity		= abstract rule shift	Dang et al., 2012
			↑ object feature shift	
	D_2_ agonist	Low DA synthesis	↑	van Holstein et al., 2011
	D_2_ antagonist		↓ longer RT	
**ATTENTIONAL SET-SHIFT**				
	D_2_ binding	Dorsal striatum	Binding reduced during shifts	Monchi et al., 2006a; Ko et al., 2009
		Anterior		
		Cingulate		
	D_2_ antagonist		↓ EDS performance	Mehta et al., 1999, 2004
			= IDS performance	
	D_2_ agonist	Methylphenidate	=	Elliott et al., 1997

= no effect, ↑ increased performance, ↓ decreased performance.

DA, dopamine; RT, reaction time; EDS, extra dimensional set-shift; IDS, intradimensional set-shift.

For DA transmission through D_1_ receptors, an optimal level of DA activity is required for best working memory performance (Williams and Goldman-Rakic, 1995; Zahrt et al., 1997; Vijayraghavan et al., 2007). Similar results were obtained for flexible responding in the WCST where impaired performance is observed for both high and low prefrontal D_1_ (but not D_2_) binding [(Takahashi et al., 2008), but see Karlsson et al. (2011)].

When receptor availability is assessed during task performance, it provides a measure of task-related release of endogenous DA. Reduced binding to D_2_ receptors in the dorsal striatum (Monchi et al., 2006a) and anterior cingulate cortex (Ko et al., 2009) during set-shifting (see Monchi et al., 2006b) suggests that DA is indeed released during tasks requiring flexibility. Transient inactivation of dorsolateral PFC activity impaired striatal DA release as well as task performance, suggesting both are under top–down control by the dorsolateral PFC (Ko et al., 2008).

Taken together, these findings indicate that DA is activated and can influence performance on set-shifting tasks through D_2_ receptors in the striatum and anterior cingulate cortex, whereas in the PFC, DA activity through D_1_ receptors can modulate performance. In addition, optimum values may exist for both extracellular DA concentrations and DA receptor numbers. The majority of studies relating performance on cognitive flexibility tasks to DA-receptor binding potential have specifically focused on binding to D_2_ receptors in specifically delineated brain areas. Therefore, although this provides evidence that D_2_ receptors modulate performance in these types of tasks, one cannot exclude the involvement of D_1_ receptors.

### Pharmacological manipulations affecting DA signaling

DA neurotransmission during task performance can be influenced by administration of pharmacological agents that directly bind to DA receptors or by drugs that induce DA release. Combining the administration of pharmacological agents with functional imaging during task performance indicates in which brain areas modulation by DA is most pronounced.

### DA antagonist

Systemic administration of the D_2_ receptor antagonist sulpiride slows response times during task-switching (Mehta et al., 2004) and impairs performance of an extra-dimensional set-shift, without affecting intra-dimensional set-shifting (Mehta et al., 1999, 2004). Sulpiride enhances performance on reward-based reversal learning (van der Schaaf et al., 2012). This behavioral effect was stronger in subjects with higher working memory capacity [which is assumed to reflect higher striatal DA synthesis capacity (Cools et al., 2008)]. In addition to behavioral effects, sulpiride also increased striatal BOLD signals during unexpected outcomes, irrespective of whether the unexpected outcome was a reward or a punishment (van der Schaaf et al., 2012).

### Indirect DA agonist

Methylphenidate is a psychostimulant that increases striatal extracellular DA levels (Volkow et al., 2001), but also affects serotonin (5-hydroxytryptamin, 5-HT) and noradrenaline (Kuczenski and Segal, 1997). Administration of methylphenidate leads to displacement of raclopride binding to D_2/3_ receptors (Clatworthy et al., 2009). These changes in the post commissural part of the caudate nucleus were associated with effects on reversal learning, such that a large displacement following methylphenidate was associated with impaired performance and a small displacement with improved performance (Clatworthy et al., 2009). As these effects may depend on individual variation in receptor availability and DA synthesis capacity, behavioral effects of the psychostimulant on measures of flexibility are likely to be averaged out when the individual variation is not taken into account—which may explain the negative results on attentional set-shifting (Elliott et al., 1997).

Administration of methylphenidate influences brain activation in ventral striatal regions during behavioral adaptation and modulates activity in frontal regions during cognitive control. Thus, activation in ventral striatal regions was reduced during reversal errors (even in the absence of behavioral effects), whereas in prefrontal regions, increased activation was observed following correct responses (Dodds et al., 2008). The balance of DA in frontal and striatal regions may therefore be crucial in regulating the balance between cognitive control and cognitive flexibility.

### DA agonist

Interestingly, DA synthesis capacity also influences the effect of direct DA agonists on task performance. While (Mehta et al., 2001) originally observed an increase in non-perseverative errors and slowed reaction times during probabilistic reversal learning after administration of the D_2_ agonist bromocriptine, Cools et al. (2009) later showed that this drug impaired reversal learning from unexpected rewards in subjects with high DA synthesis capacity, but improved the same parameter in subjects with low synthesis capacity in striatal regions.

The beneficial effect of D_2_ receptor stimulation in subjects with low DA synthesis capacity is not limited to reversal learning. Bromocriptine can also improve performance on the WCST (Kimberg et al., 1997) and task-switching performance (van Holstein et al., 2011) in subjects with low DA synthesis capacity, whereas no effects are observed following administration of pergolide, which differs from bromocriptine in that it also activates D_1_ receptors (Kimberg and D'Esposito, 2003). That the improvement on task switching after bromocriptine can be specifically related to the function of D_2_ receptors was shown by (van Holstein et al., 2011), as pre-treatment with the D_2_ antagonist sulpiride blocked the beneficial effect. Therefore, performance of subjects with high DA synthesis capacity is impaired following administration of bromocriptine, and increases following administration of sulpiride.

### Summary and conclusion

To conclude (see Table 1), flexible updating of behavior in set-shifting tasks (WCST and attentional set-shifting) as well as task switching is associated with increased DA neurotransmission through D_2_-receptors. In particular, the mediating effects of D_2_ signaling on task performance have been observed in the dorsal striatum and anterior cingulate cortex, which is in line with observations from imaging and lesion studies suggesting the involvement of the connections between PFC and dorsal striatum in the regulation of these types of flexibility (Owen et al., 1991; Sohn et al., 2000). This also concurs with observations in patients with PD. In the early stages of PD, when DA depletion is largely limited to the dorsal striatum, patients show impairments in task switching whereas reversal learning performance is spared. Administration of levodopa reverses the impairments in task switching, whilst it impairs performance on reversal learning probably due to overstimulation of DA receptors in ventral striatal regions (Cools, 2006; Kehagia et al., 2010). In control subjects increased D_2_-mediated transmission also impairs reversal learning, although this may turn into an improvement when DA synthesis capacity is low.

Human studies have particularly shown the importance of individual differences in the DA system. Individual differences in DA synthesis capacity influence both task performance and effects of manipulations to the DA system in different types of flexibility. Individual differences in D_2_ receptor availability also influence stimulation-induced changes in performance during reversal learning. The combined study of manipulations to the DA system with performance on behavioral tasks, indicate that DA transmission in the ventral striatum changes during reversal learning.

These results also indicate that there may be differences in the involvement of DA in reversal learning compared to set-shifting and task switching. As noted before, these paradigms are thought to represent different levels of complexity and may depend on different brain areas. However, studies differ in the task designs used to study one type of cognitive flexibility. Therefore, replication of effects of DAergic manipulations using similar task designs would help in delineating the possible differences in DA contribution to reversal, set-shifting and task switching.

A question remains in what way D_1_ receptors contribute to behavioral performance during cognitive flexibility tasks. Direct manipulations of D_1_ signaling or studies relating performance on behavioral task to D_1_ receptors availability are scarce. Combining the administration of pharmacological agents with functional imaging during performance of different behavioral paradigms may provide more insight on the effects of DA on cognitive flexibility in prefrontal and striatal regions.

## Pharmacological manipulations in animals

The use of pharmacological imaging in human subjects provides insight into the role of DA in cognitive flexibility, but the use of animals permits direct (and invasive) manipulations and measurements and can extend and specify findings obtained in human subjects. Here, we will discuss animal studies that have used pharmacological manipulations of the DA system or DA depletion to investigate in what way DA in prefrontal and striatal regions contributes to cognitive flexibility.

### DA depletion studies

In rodents, lesioning DAergic projections in the nucleus accumbens core (though DA in the medial PFC was similarly affected) impairs both spatial discrimination and reversal learning on a T-maze (Taghzouti et al., 1985). Selective depletion of DA neurotransmission in the dorsomedial striatum impairs odor guided reversal learning, without affecting initial discrimination learning (O'Neill and Brown, 2007). A selective deficit in reversal learning following DA depletion in the dorsomedial striatum was observed in primates as well (Clarke et al., 2011). The deficit in reversal learning following DA depletion is not perseverative, suggesting that DA may be particularly important for the learning phase after reversal, rather than mediating response inhibition to the previously rewarded side. The effect was not only shown in the first, but also in subsequent reversals. Importantly, the deficit is neurochemically specific, as depletion of 5-HT neurotransmission in the mediate caudate nucleus does not affect behavioral performance during reversal learning (Clarke et al., 2011). A previous study also found decreased performance on reversal learning (although this did not reach significance) (Collins et al., 2000). Subsequently, Crofts et al. (2001) showed that although acquisition, maintenance and initial shifting of an attentional set are intact, monkeys with DA depletion in the caudate are impaired when they have to make an attentional shift to a stimulus dimension that was learned to be irrelevant in a previous extra dimensional shift (Collins et al., 2000; Crofts et al., 2001). Therefore, DA in the caudate nucleus appears to be involved in situations that require a shift of established cognitive sets (Collins et al., 2000).

In contrast to DA depletion in striatal regions, selective DA depletion in frontal regions is complicated by the accompanied depletion of noradrenaline (Roberts et al., 1994; Crofts et al., 2001). Although Roberts et al. (1994) observed a specific improvement in performance on extra-dimensional set-shifts after prefrontal catecholamine depletion in non-human primates, a later study suggests that this may actually result from an inability to maintain an attentional set (Crofts et al., 2001). Prefrontal catecholamine depletion is associated with long lasting enhancement of striatal DA release, suggesting that it may be the balance between DA levels in prefrontal and striatal regions rather than DA levels in either region that affects behavior (Roberts et al., 1994).

### DA versus 5-HT

Based on data from depletion studies, a neurochemical dissociation between prefrontal and striatal regions in the control of cognitive flexibility during reversal learning has been suggested. In the caudate nucleus, DA, but not 5-HT depletion impairs performance during reversal learning. Previously, it was reported that 5-HT, but not DA neurotransmission in the OFC is required for successful behavioral adaptation in a spatial reversal learning task (Clarke et al., 2004, 2007). Depletion of 5-HT in the OFC specifically impairs reversal learning by increasing perseverative responding, but does not affect attentional set-shifting (Clarke et al., 2005). OFC DA depletion, however, leads to impaired extinction, albeit not in a perseverative manner (Walker et al., 2009). The contributions of 5-HT and DA neurotransmission to cognitive flexibility therefore appear to be confined to separate functions related to regions of the cortico-striatal circuit. Recently, (Groman et al., 2013) suggested that the balance between 5-HT levels in the OFC and DA levels in the dorsal striatum contributes to individual differences in cognitive flexibility. Reduced performance on a reversal learning task is associated with low levels of 5HT in the OFC when DA levels in the putamen are low, but not when DA levels in the putamen are high (Groman et al., 2013). These findings indicate that cognitive flexibility is under control of DA and 5-HT, while other data show involvement of noradrenaline, as well (Bouret and Sara, 2004; Lapiz and Morilak, 2006; Seu et al., 2009).

### Effects of psychostimulants

Psychostimulants such as methylphenidate, (meth)amphetamine and cocaine increase release of DA and other monoamines by blocking catecholamine re-uptake or promoting DA release (Sulzer et al., 2005). Administration of methylphenidate in rodents does not affect reversal learning (Seu and Jentsch, 2009; Cheng and Li, 2013), although the latter authors observed beneficial effects in animals with reversal learning impairments (spontaneously hypertensive rats). Effects of amphetamine and methamphetamine on reversal learning have been variable, but possibly dose-dependent: high doses (5 mg/kg) impair reversal learning (Ridley et al., 1981; Arushanian and Baturin, 1982; Idris et al., 2005; Cheng et al., 2007; White et al., 2009; Izquierdo et al., 2010; Kosheleff et al., 2012; Talpos et al., 2012), while intermediate doses 1–2 mg/kg show no effect or improved learning (Wilpizeski and Hamilton, 1964; Kulig and Calhoun, 1972; Mead, 1974; Weiner and Feldon, 1986; Weiner et al., 1986; Daberkow et al., 2008; Pastuzyn et al., 2012; Soto et al., 2012) and low doses again impair reversal performance (Ridley et al., 1981; Idris et al., 2005). These results are compatible with the general idea that cognitive function depends on DA activity in an inverse U-shaped fashion (Cools and D'Esposito, 2011; Arnsten et al., 2012). However, given the multiple and differential effects of psychostimulants on monoamine release in prefrontal and striatal regions it is often difficult to conclude whether these effects depend on increased DA release. Yet, for methylphenidate Cheng and Li (2013) showed that the beneficial effect were blocked by local injections with haloperidol in the OFC.

### Systemic effects of DA (ANT)agonists

While selective depletion studies indicate specific brain areas where DA modulates flexible behavior, administration of pharmacological agents that are selective for a specific receptor subtype indicate how D_1_ and D_2_ receptor subtypes are involved. In primates, both stimulation and inhibition of D_2_/D_3_ receptor function results in difficulties in adapting behavior following changing task demands, but not during acquisition of the original discrimination (Smith et al., 1999; Lee et al., 2007). Administration of the D_2_/D_3_ antagonist raclopride affects performance on reversal learning when administered alone, but only when the reversal is preceded by retention of the originally acquired discrimination (Lee et al., 2007). Performance is also reduced by the D_3_/D_2_ agonist 7-OH-DPAT (Smith et al., 1999) and this deficit is antagonized by co-administration with the D_2_/D_3_ antagonist raclopride, but not the D_2_-selective antagonist sulpiride, suggesting stimulation of D_3_ receptors impairs performance (Smith et al., 1999).

In rodents, like in primates, administration of a D_2_/D_3_ agonist (quinpirole) impaired spatial reversal learning in an operant chamber by increasing the number of perseverative errors. Administration of a D_2_/D_3_ antagonist (raclopride) or selective D_3_ antagonist (nafadotride) had no effect (Boulougouris et al., 2009). The quinpirole-induced deficit is attenuated when raclopride is co-administered, but worsens after co-administration with nafadotride. Selective stimulation of D_2_-receptors (co-administration of quinpirole and nafadotride) increased both the number of discrimination errors and of perseverative and learning errors in the reversal phase (Boulougouris et al., 2009). Thus, stimulation of D_3_ receptors may be important for the acquisition of altered response-reward contingencies during reversal learning whereas D_2_-receptor activation may cause a more generalized impairment (Boulougouris et al., 2009).

Systemic administration of a D_1_/D_5_ antagonist does not affect reversal learning in primates (Lee et al., 2007), though in rodents systemic administration of a D_1_ agonist (SKF-812979) impairs early, but not late stages of reversal learning (Izquierdo et al., 2006). Extradimensional set-shifting on the other hand improves following intermediate, but not high or low doses of a D_1_ agonist (Nikiforuk, 2012).

These findings suggest that D_2_-like receptors contribute to the regulation of cognitive flexibility, possibly in a dose-dependent manner. System administration of D_1_-like receptors has received less attention and could affect cognitive flexibility depending on the species or behavioral task used.

### Local effects in the striatum

Local manipulations of DA neurotransmission can elucidate in which way DA neurotransmission in specific subregions of the fronto-striatal circuit can contribute to cognitive flexibility (although see, Arnt, 1985) for the limitations of this approach). Execution or suppression of actions leading to reward are controlled by two parallel cortico-striato-thalamo-cortical pathways (Frank and Claus, 2006). From the striatum, output neurons in the *direct pathway* connect to cortical regions via connections to globus pallidus pars interna (GPi)/substantia nigra pars reticulata (SNr) and thalamus. Output neurons in the *indirect pathway* project via globus pallidus pars externa, subthalamic nucleus to GPi/SNr, thalamus and cortex. Activity in these pathways can be differentially modulated by activation of D_1_ or D_2_ receptors in the striatum (Frank and Claus, 2006). Yawata et al. (2012) investigated pathway specific control of reward learning and cognitive flexibility. Blocked neurotransmission in the direct pathway, combined with D_1_ blockade in the contralateral nucleus accumbens impaired the acquisition phases of the original discrimination as well as the discrimination presented after a reversal or a rule shift, while stimulation of D_1_ receptors did not influence behavior (Yawata et al., 2012). Application of a D_2_ agonist combined with contralateral blockade of the indirect pathway induced perseverative responding during reversal learning and also affected rule shifting, without affecting acquisition of the original discrimination problem (Yawata et al., 2012). These findings suggest that within the nucleus accumbens, stimulation of DA D_1_ receptors (direct pathway) aids the acquisition and relearning of behavioral responses to a particular stimulus, whereas suppression (i.e., a phasic interruption) of D_2_-mediated transmission (indirect pathway) may be required to allow reorganization of ongoing behavioral patterns. These results are in line with previous findings reporting impaired reversal learning after local stimulation of D_2_ receptors, while during set-shifting blocking D_1_ receptors impaired maintenance of the new strategy and stimulation of D_2_ receptors induced perseverative responding (Haluk and Floresco, 2009).

### Local effects in the prefrontal cortex

DA depletion in the OFC did not affect reversal learning (Clarke et al., 2007), but local manipulation of DA receptors in the OFC can influence aspects of cognitive flexibility. Blockage of D_1_ or D_2_ receptors in OFC prevents development of discriminative reaction times to high and low rewards under reversal conditions, without affecting accuracy (Calaminus and Hauber, 2008). In a task that required rats to adapt behavior following a change in reward value, by manipulating the amount of lever presses required to obtain a food pellet, local inhibition of D_1_ but not D_2_ receptors in the OFC impaired performance (Winter et al., 2009). In the MPFC, local inhibition of both D_1_ and D_2_ receptors inhibits performance (Winter et al., 2009). Set-shifting ability in a maze-based shifting task is affected by manipulations of several DA receptors in the MPFC. Local blockade of D_1_ and D_2_ receptors as well as stimulation of D_4_ receptors results in perseverative responding, whereas blockade of the D_4_ receptor improves performance (Ragozzino, 2002; Floresco et al., 2006b). This contrasts with the findings of D_1_ blockade in the nucleus accumbens, which did not induce perseverative responding, but affected maintenance of the new strategy.

### *In vivo* DA measurements related to cognitive flexibility

Only a few reports on the measurement of extracellular levels of DA in the brain (reflecting DA release) are available. In the nucleus accumbens, these levels are higher during acquisition of a rule shift compared to simple rule acquisition in a T-maze set-shift paradigm (Stefani and Moghaddam, 2006), clearly suggesting a role for DA in the nucleus accumbens in the regulation of cognitive flexibility, in particular strategy or set-shifting. In the mPFC, both rule acquisition and rule shifting in a T-maze are accompanied by increased DA levels and higher basal mPFC DA levels were associated with rapid shifting between discrimination rules (Stefani and Moghaddam, 2006). After inhibiton of COMT, animals also show increased task-related, but not basal extracellular DA levels in the medial PFC, suggesting that task-induced increases in PFC DA release may contribute to set-shifting performance (Tunbridge et al., 2004).

DA (but not noradrenaline) release in the MPFC is elevated and prolonged during performance of a spatial reversal session in a skinnerbox, compared to release in a discrimination session preceding reversal (van der Meulen et al., 2007). Within the reversal session, the DA elevation was most pronounced during the phase in which rats improved performance.

These findings suggest elevated DA release in both striatal and prefrontal regions during execution of cognitive flexibility tasks.

### Summary and conclusion

Taken together (see Table 2), DA appears to be actively involved in the performance of tasks requiring cognitive flexibility: DA release is increased, local DA depletion impairs performance and pharmacological interference alters task execution. Whereas DA depletion studies indicated ventral and dorsomedial striatum as the primary location where DA influences cognitive flexibility, specific DA receptor stimulation/blockade studies and *in vivo* release measurements implicate prefrontal regions as well. A complicating factor is that manipulation of prefrontal DA also affects striatal DA transmission (Roberts et al., 1994).

**Table 2:** **Summary of effects of pharmacological manipulations to the dopamine system on cognitive flexibility in animals**.

**Paradigm**	**Region**		**Manipulation**	**Performance**	**References**
**SET-SHIFT**					
	Nucleus accumbens	D_1_	Agonist	=	Haluk and Floresco, 2009
			Antagonist	↓ Impaired maintenance new strategy	
		D_2_	Agonist	↓ perseveration	Haluk and Floresco, 2009
			Antagonist	=	
	Dorsomedial striatum		Depletion	↓ EDS, only when switching to previously dimension	Collins et al., 2000
	MPFC	D_1_	Agonist	=	Floresco et al., 2006b
			Antagonist	↓ Perseverative	Ragozzino, 2002
		D_2_	Agonist	=	Floresco et al., 2006b
			Antagonist	↓ more trials/errors to criterion. Perseverative	
		D4	Agonist	↓ more trials/errors to criterion. Perseverative	Floresco et al., 2006b
			Antagonist	↓ more trials/errors to criterion. Perseverative	
	Frontal		Depletion	↓ Maintenance of set (IDS)	Crofts et al., 2001
					Roberts et al., 1994
**REVERSAL**					
	Systemic (primate)	D_1_	Antagonist	=	Lee et al., 2007
	Systemic (rodent)			↓	Izquierdo et al., 2006
	Systemic (rodent)	D_2_/D_3_	Agonist	↓ perseveration	Boulougouris et al., 2009
	Systemic (primate)	D_2_/D_3_	Antagonist	↓ more trials/errors to criterion	Lee et al., 2007
	Systemic (rodent)			=	Boulougouris et al., 2009
	Systemic (primate)	D_3_/D_2_	Agonist	↓ more trials/errors to criterion	Smith et al., 1999
	Nucleus accumbens	D_1_	Agonist	=	Haluk and Floresco, 2009
			Antagonist	=	Calaminus and Hauber, 2007
		D_2_	Agonist	↓ trials to criterion/errors, but not perseveration	Haluk and Floresco, 2009
			Antagonist	=	Calaminus and Hauber, 2007
			Depletion	↓	Taghzouti et al., 1985
	Dorsomedial striatum		Depletion	↓ more trials to criterion	O'Neill and Brown, 2007
					Clarke et al., 2011
	OFC	D_1_	Antagonist	↓ absence discriminative reaction times (high/low reward)	Calaminus and Hauber, 2008
					Winter et al., 2009
				↓ impaired maintenance low effort response	
		D_2_	Antagonist	↓ absence discriminative reaction times (high/low reward)	Calaminus and Hauber, 2008
					Winter et al., 2009
				= reversal required effort not affected	
	MPFC	D_1_	Antagonist	↓ impaired maintenance low effort response	Winter et al., 2009
		D_2_	Antagonist	↓ impaired maintenance low effort response	Winter et al., 2009

= no effect, ↑ increased performance, ↓ decreased performance.

EDS, extra dimensional set-shift; IDS, intradimensional set-shift; MPFC, medial prefrontal cortex; OFC, orbitofrontal cortex.

It is important to note that impairment of reward-related learning and cognitive flexibility following perturbations in DA signaling is almost always of transient nature: subjects eventually do make the switch when sufficient trials are presented, suggesting that DA may facilitate these behaviors, but is not indispensable.

Interestingly, most pharmacological studies investigating the involvement of DA-subtype selective receptors have indicated that striatal blockade of D_1_-receptors and overactivation of D_2_-receptors impairs performance. This was most elegantly shown in the study of Yawata et al. (2012): DA signaling through D_1_ receptors in the nucleus accumbens and the direct basal ganglia pathway contributes to the acquisition of a new reward-directed behavior in a four-armed maze once switching has occurred (i.e., D_1_ stimulation could contribute to new learning following a behavioral switch), whereas suppression of D_2_-mediated transmission in the accumbens and the indirect pathway is required for the reorganization of behavioral patterns. A transient elevation in DA potentiates connections in the direct pathway to initiate movement toward reward, whereas a transient dip in DA potentiates connections in the indirect pathway to suppress movements that are no longer rewarded (Hong and Hikosaka, 2011). The findings from animal studies do indicate a role for the DA in the nucleus accumbens mediating cognitive flexibility, both reversal and strategy or set-shifting, whereas less research has focused on local manipulation of D_1_ or D_2_ receptors in dorsomedial or dorsolateral striatal regions. However, a role for dorsal striatal regions has been indicated by selective DA depletion studies as well as a significant amount of human data. Moreover, in the primate dorsal striatum (caudate and putamen), availability of D_2_-receptors can be related to performance during reversal but not discrimination learning (Groman et al., 2011). This warrants further investigation of the effects of manipulating D_1_ or D_2_ signaling in striatal regions other than the nucleus accumbens.

In general, these conclusions are similar to those based on human data, as discussed in the previous section. However, unlike what was reported in humans, D_2_-based manipulations seem to affect lower order (cue reversal) and higher order (rule or task switch) processes in a similar way. It is unclear if D_2_-mediated effects in animals depend on DA synthesis capacity.

## Contributions of DA genotype to cognitive flexibility in humans

Individual variability in executive functioning may be subserved by a strong genetic component (Friedman et al., 2008). The expression of complex traits such as cognitive flexibility is likely regulated by multiple genes that each contribute a small effect. Several polymorphisms in genes affecting DA functioning have been investigated to explain individual variability in cognitive flexibility.

### DA receptors and intracellular signaling

#### D_1_

DARPP-32 (DA and cAMP regulated phosphoprotein of 32kDA) is strongly expressed in medial spiny neurons in the striatum, where it is stimulated by D_1_ and inhibited by D_2_ receptor activation and mediates post-receptor effects of DA (Nishi et al., 1997; Svenningsson et al., 2004). Enhanced performance on several cognitive tasks, including the WCST, was observed for a frequent haplotype in the DARPP-32 gene that is associated with increased post-mortem DARPP-32 expression and affects structural and functional connectivity between PFC and striatum (Meyer-Lindenberg et al., 2007). The polymorphism was also associated with better learning from positive feedback (Frank et al., 2007). This suggests D_1_ receptors in the striatum could contribute to learning after positive feedback, supporting successful switching of behavior in cognitive flexibility tasks by maintaining responses to the newly rewarded site.

#### D_2_

The DRD2-TAQ1 polymorphism is located close to the exon coding for the D_2_ receptor. A1-allele carriers show a reduced number of available D_2_ receptors [(Thompson et al., 1997; Pohjalainen et al., 1998), but see Lucht and Rosskopf (2008)] and the A1-allele is associated with increased DA synthesis in the striatum (indicating reduced autoreceptor-mediated feedback regulation) (Laakso et al., 2005). In a probabilistic learning task, carriers of the A1-allele showed reduced ability to learn from errors accompanied by functional changes in the frontostriatal circuitry (Klein et al., 2007). A1-carriers showed blunted reward-related activity in the NAC, reduced activity in the posterior medial frontal cortex during negative feedback and reduced interactions between the medial frontal cortex and hippocampus (Klein et al., 2007). The use of feedback is required to adapt responding during reversal learning and, not surprisingly, A1-carriers perform worse (Jocham et al., 2009). Following presentation of a reversal, they were less likely to maintain the newly rewarded response, but kept alternating responses and showed diminished activation of orbitofrontal and ventral striatal regions during reversals (Jocham et al., 2009). Task-switching performance on the other hand is improved in A1-carriers, who show reduced switch costs associated with decreased activity in the lateral PFC and decreased connectivity between PFC and dorsal striatal regions (Stelzel et al., 2010). Switching tasks does not depend on the use of feedback and is supported by different circuits/areas than switching responses based on the use of feedback (Stelzel et al., 2010). This illustrates how impaired DA transmission could have different effects depending on the operationalization of the cognitive flexibility task that is used, i.e., whether on-line feedback-induced response adaptation (“learning”) is essential or not.

A second polymorphism affecting availability of striatal D_2_ receptors is the C957T polymorphism of the DRD2 gene (Hirvonen et al., 2004, 2005). CC-allele carriers show reduced binding potential to striatal D_2_ receptors (Hirvonen et al., 2004, 2005) and impaired responding in the WCST (Rodriguez-Jimenez et al., 2006). In addition, CC-allele carriers are reduced in their ability to use negative feedback in a probabilistic reinforcement learning task (Frank et al., 2007). These concurrent findings suggest that reduced availability of D_2_ receptors is associated with impaired cognitive flexibility, resulting from an inability to use negative feedback to adapt behavior.

### DA transporter and metabolizing enzymes

The DA transporter (DAT) regulates re-uptake of DA from the synaptic cleft in striatal regions, whereas its influence in the PFC is less pronounced (Sesack et al., 1998). Using a task-switching protocol based on the WCST, Garcia-Garcia et al. (2010) observed impaired performance and electrophysiological differences in 9-repeat allele carriers compared to 10-repeat allele carriers of the DAT gene. During task-switching, manipulation of reward anticipation affects performance and striatal activity depending on DAT genotype, suggesting striatal DA levels mediate the influence of motivational effects on cognitive flexibility (Aarts et al., 2010). However, considering that it is unclear how this polymorphism relates to DAT expression *in vivo* [Heinz et al., 2000; Martinez et al., 2001; van Dyck et al., 2005; van de Giessen et al., 2009; meta-analysis by Costa et al., 2011], these results should be interpreted with caution.

The polymorphism that has received most attention relating DAergic gene function to executive functioning is the Valine (Val)/Methione (Met) polymorphism at codon 158 of the Catechol-*O*-methyltranserase (COMT) gene (Lotta et al., 1995). Activity of COMT is thought to be lower in homozygote Met allele carriers compared to homozygote Val carriers, presumably resulting in higher prefrontal DA levels in Met homozygotes (Lotta et al., 1995; Chen et al., 2004; Meyer-Lindenberg et al., 2005), although striatal DA levels may also be altered (Akil et al., 2003). Most studies investigating the association between the COMT Val/Met polymorphism and cognitive flexibility used perseverative responding or perseverative errors in the WCST as a measure of flexible behavior. Results have not been consistent: although an initial meta-analysis (Barnett et al., 2007) reported a small effect of COMT genotype on performance in the WCST, with reduced perseverative errors for the Met homozygotes, a second meta-analysis could not confirm an association between COMT genotype and perseverative responding on the WCST and several other cognitive measures, suggesting that the COMT polymorphism does not consistently relate to cognitive functioning (Barnett et al., 2008). It has been suggested that the variety of cognitive functions contributing to WCST performance complicate attribution of impaired performance to deficits in cognitive flexibility or deficits in cognitive stability (Bilder et al., 2004). Other test measures of cognitive flexibility might be more sensitive and more selective indicators of alterations in this function.

Despite the inconsistent effects of COMT genotype on perseverative errors in the WCST, the COMT Val/Met genotype is associated with differential activation patterns in the PFC during other cognitive paradigms (Mier et al., 2010). Therefore, it is interesting to relate COMT genotype to neural activation during other tasks that measure separate aspects of cognitive flexibility more specifically, to see whether this genotype influences neural activation in these tasks. Indeed, when (Krugel et al., 2009) studied the influence of COMT gene polymorphisms on performance and neural activity during probabilistic reversal learning, Val homozygotes performed better than Met homozygotes and showed increased striatal BOLD responses during prediction errors. In addition, higher connectivity between frontal and ventral striatal regions could be related to learning rate in Val homozygotes (Krugel et al., 2009). Interestingly, these findings suggest that striatal activity reflecting prediction errors might be modulated by DA levels in the PFC. However, during acquisition of probabilistic reinforcement learning, Val homozygotes show reduced switching of responses following negative outcomes on a trial-by-trial basis (Frank et al., 2007). This suggests that striatal DA function may be differentially regulated by DA levels in the PFC during response acquisition or adaptation of an existing response. In addition to a behavioral advantage during reversal learning, Val homozygotes also have smaller switch costs on a task switching paradigm when trials have short intervals (Colzato et al., 2010). Together these findings indicate a behavioral advantage on both reversal learning and task switching paradigms for Val homozygotes, suggesting that lower baseline levels of prefrontal DA may benefit cognitive flexibility in humans.

### Summary and conclusion

A substantial amount of studies investigating the influence of genes mediating DA function on cognitive flexibility have limited analysis to a task that likely measures several complex cognitive functions, i.e., the WCST (Friedman et al., 2008). A more promising approach may be to study the effect of DA related genes on well-defined operationalizations of cognitive flexibility, such as initial discrimination learning, reversal learning, attentional set-shifting or task switching. A confound in the study of cognitive effects of genetic polymorphisms is that the effect of a polymorphism on DA transmission or even on gene expression is often not known. This hampers translational approaches, in which effects of increased or decreased expression and/or DA transmission might be studied in a controlled and reproducible manner.

To summarize, the studies reviewed above suggest an association between polymorphisms regulating DA function and cognitive flexibility. Reduced availability of D_2_ receptors, presumably affecting striatal DA activity, impairs the use of negative feedback and the maintenance of a new response during reversal learning and set-shifting (in the WCST), whereas increased availability of D_2_ receptors impairs task switching, suggesting different involvement of D_2_ receptors in these tasks. Striatal D_1_ signaling, mediated by DARPP-32 function, also contributes to cognitive functioning, although this has not yet been verified using specific measures of cognitive flexibility. Presumed lower levels of prefrontal DA, mediated by COMT-genotype appear to facilitate behavioral adaptation in both reversal learning and task-switching paradigms (see Table 3).

**Table 3:** **Effects of polymorphisms in dopamine related genes on cognitive flexibility in human subjects**.

**Paradigm**	**Gene**		**Presumed DA effect**	**Performance**	**References**
**REVERSAL**					
	D_2_	A1	↓ D_2_ binding striatum	↓ reversal learning	Jocham et al., 2009
	COMT	Val/Val	↓ COMT activity PFC	↑ reversal learning	Krugel et al., 2009
**TASK SWITCH**					
	D_2_	Non-A1	↑ D_2_ binding striatum	↓increased switch cost	Stelzel et al., 2010
	DAT	9-repeat	Striatum	↓increased RT cue switch/	Garcia-Garcia et al., 2010
				= task switch	Aarts et al., 2010
				↑ task switch high rewarded trials	
	COMT	Val/Val	↓ COMT activity PFC	↑ reduced switch cost	Colzato et al., 2010
**WCST**					
	D_2_	C957T – CC	↓ D_2_ binding striatum	↓ WCST categories completed, perseveration	Rodriguez-Jimenez et al., 2006
	COMT	Val/Val	↓ COMT activity PFC	=	Barnett et al., 2008
	DARPP-32	Haplotype	Striatum	↑ WCST performance	Meyer-Lindenberg et al., 2007

= no effect, ↑ increased performance, ↓ decreased performance.

PFC, prefrontal cortex; RT, reaction time; WCST, Wisconsin Card Sorting Task.

To conclude, considering that the genetic underpinnings of complex cognitive functions are likely to be polygenic and not limited to DA, studying additive genetic effects of DA related genes on cognitive flexibility as well as the study of interactions between DA related genes and other genes regulating frontostriatal function could provide a better understanding of the genetic basis of cognitive flexibility (Frank and Fossella, 2011).

## Effects of genetic manipulations in DA related genes on cognitive flexibility in animals

The use of genetically modified animals provides an invaluable tool to study the role of DA related genes in cognitive flexibility. Selectively targeted mutations on a known genetic background can elucidate the genetic and neurobiological basis of complex behavior.

### DA deficiency

An example of an advanced genetic approach is selective reinstatement of DA signaling in ventral or dorsal striatum of DA-deficient mice (Darvas and Palmiter, 2011). Restoring DA signaling specifically to either dorsal or ventral striatum supports acquisition and reversal of a turn-based escape strategy in a water maze (Darvas and Palmiter, 2011). However, the ability to switch from one escape strategy to another (strategy set-shift) is impaired when DA signaling is limited to the ventral striatum, suggesting DA neurotransmission in the dorsal striatum is required for strategy set-shifting, whereas DA in either ventral or dorsal striatum is sufficient to support reversal learning (Darvas and Palmiter, 2011). It should be noted, however, that the translational value of the tasks used is not established.

### DA receptors and intracellular signaling

#### D_1_

Mice lacking functional D_1_ receptors show attenuated operant responding for reward (El-Ghundi et al., 2003). They show a general deficit in reinforcement learning, impaired motivation to work for a reward, are slow to discriminate between a reinforced and non-reinforced lever and are impaired in reversal learning, during which they maintain responding to both levers. Heterozygote mice are also impaired on reversals, although not as severely (El-Ghundi et al., 2003). The observed general deficits in motivation and reinforcement learning in D_1_-knockout mice, however, prevent the drawing of conclusions about the contribution of D_1_ receptors to cognitive flexibility.

Activation of D_1_ receptors modulates striatal function through phosphorylation of DARPP (Walaas and Greengard, 1984). Next to a minor reduction in performance during discrimination learning, DARPP-32 knockout mice show a pronounced deficit in reversal learning. Although knockout mice eventually were able to switch responding to the newly rewarded side, it took them significantly more sessions to do so (Heyser et al., 2000). This is indirect evidence that D_1_ receptor activation is needed for reversal learning.

#### D_2_

Genetic manipulations of D_2_ receptors also affect performance on cognitive flexibility tasks. Female mice with a complete knock-out of functional D_2_ receptors make more errors during odor discrimination and reversal learning whereas male D_2_-knockouts are impaired during reversal learning only; both sexes show perseveration to the previously rewarded side (Kruzich and Grandy, 2004; Kruzich et al., 2006). This was confirmed by De Steno and Schmauss (2009), who also showed a similar impairment with chronic treatment with the D_2_ antagonist haloperidol. Glickstein et al. (2005) observed a deficit of male D_2_-knockouts during compound discrimination, but not reversal, whereas D_3_ receptor knockouts showed increased performance during the reversal. The differences in behavioral performance were paralleled by opposite prefrontal activation patterns following the task sequence: activity dependent gene expression in the MPFC is increased for D_3_ mutants and decreased for D_2_ mutants (Glickstein et al., 2005; De Steno and Schmauss, 2009). Interestingly, knockout of neither D_2_ nor D_3_ receptors affects performance on intra- or extradimensional set-shifts (De Steno and Schmauss, 2009), suggesting differential contribution of D_2_/D_3_ receptors to the regulation of reversal learning or set-shifting.

Selective overexpression of D_2_ receptors in the striatum does not affect learning of a discrimination, a reversal or an intra- or extradimensional set-shift. Response latencies were longer during reversal trials only, suggesting the animals had some difficulties adapting established responses (Kellendonk et al., 2006). Interestingly, these mice also show physiological changes in the medial PFC where DA turnover was decreased and activation of D_1_ receptors increased (Kellendonk et al., 2006).

### Metabolizing enzymes

Overexpression of the human COMT-Val polymorphism in mice increases COMT enzyme activity (suggesting lower prefrontal extracellular DA) and induces specific deficits in cognitive flexibility. Although discrimination and reversal learning are not affected, these mice make more errors and need more time to complete an extra-dimensional set-shift (Papaleo et al., 2008). In contrast to behavioral impairments observed after increased COMT enzyme activity, pharmacological inhibition of COMT can improve performance (Tunbridge et al., 2004).

### Summary and conclusion

The studies using selective DA-reinstatement in DA-deficient mice show that higher order flexibility [strategy shifting (Wise et al., 1996)] is associated with dorsal striatal DA, whereas lower order flexibility (reversal learning) may be supported by DA in all striatal areas. Similarly, human studies suggest influence of DA genotype on activity in ventral striatal regions or increased connectivity between PFC and ventral striatum during reversal learning and in dorsal striatal regions during task switching.

The D_1_ receptor is involved in cognitive flexibility, although this is overshadowed by a general impairment in goal-directed behavior in full knock-outs. DARPP-32 expression (reflecting D_1_ activity) is associated with cognitive performance in both humans and animals.

The findings described above, and the observation that performance of reversal learning in mice covaries with D_2_ receptor levels in the ventral midbrain (Laughlin et al., 2011), indicate the importance of D_2_ receptors for flexible behavior, specifically in a situation where response-reward contingencies are reversed (see Table 4). This compares to the influence of polymorphisms in the D_2_ receptor gene on the ability to learn from negative feedback in human subjects.

**Table 4:** **Effects of genetic manipulations to dopamine related genes on cognitive flexibility in animals**.

**Paradigm**	**Gene**		**Performance**	**References**
**DISCRIMINATION**				
	D_1_ KO		↓ more errors	El-Ghundi et al., 2003
	D_2_ KO	Female	↓ more errors	Kruzich and Grandy, 2004
	D_2_ KO	Male	=	Kruzich et al., 2006
	COMT-Val overexpression		=	Papaleo et al., 2008
**REVERSAL**				
	D_1_ KO		↓ more errors	El-Ghundi et al., 2003
	D_2_ KO	Male + female	↓ more errors ↓ increased RT reversal phase set-shift = reversal phase set-shift	Kruzich and Grandy, 2004 Kruzich et al., 2006
	DARPP-32 KO		↓ more errors	Heyser et al., 2000
**ATTENTIONAL SET-SHIFT**				
	D_2_ KO		=	De Steno and Schmauss, 2009 Glickstein et al., 2005
	D_2_ overexpression	Striatum only	=	Kellendonk et al., 2006
	COMT-Val overexpression		↓ impaired EDS	Papaleo et al., 2008

= no effect, ↑ increased performance, ↓ decreased performance.

KO, knock out; RT, reaction time; EDS, extradimensional set-shift.

Expressing the human COMT-Val polymorphism (increasing COMT-activity and presumably decreasing extracellular prefrontal DA) in mice impairs extra dimensional set-shift. This concurs with the improved set-shifting performance after COMT-inhibition in rats. However, presence of the Val-polymorphism in humans has been associated with a behavioral advantage during reversal learning and task-switching suggesting that confirmation of these studies is needed before we can draw conclusions.

Caution should be exerted when interpreting results from animals in which a receptor is completely knocked out as compensatory mechanisms (such as increased neurotransmitter levels) during development may contribute to the observed deficits. Also, in the case of complete knock-outs it is not possible to locate the neurobiological substrate of the impairment as the knock-out is present throughout the brain. Finally, mice with intermediate expression of specific receptors (heterozygotes) are useful for studying gene-dosage effects on behavior, which could be particularly relevant when compared to differences in receptor expression levels observed in humans.

## OCD

OCD is a psychiatric disorder that is characterized by recurrent intrusive, unwanted thoughts (obsessions) that are often accompanied by repetitive ritualistic behaviors (compulsions). Although the precise neurobiological substrates underlying OCD symptoms are not known, structural and functional imaging studies show alterations in frontal and orbitofrontal cortices and basal ganglia in OCD patients (Pujol et al., 2004; Menzies et al., 2008a,b; van den Heuvel et al., 2009; Rotge et al., 2010). Symptom severity correlates with increased functional connectivity between OFC and striatal regions (Harrison et al., 2009), which normalizes after treatment (Figee et al., 2013).

The repeated performance of ritual-like action sequences has led to the hypothesis that decreased cognitive flexibility or increased habitual behavior (Gillan et al., 2011) is a major underlying factor of OCD and could be a potential endophenotype for the disorder (Robbins et al., 2012). This might be an attractive suggestion considering that associated circuits and neurotransmitters related to these processes are (partly) known. Indications for abnormal flexibility have been described in OCD patients (Chamberlain et al., 2006; Gu et al., 2008) and there is evidence for altered DA signaling (Denys et al., 2004a,b; Moresco et al., 2007; Perani et al., 2008). Therefore, an important question is how DA contributes to this disorder. In the next sections, we will describe studies reporting alterations in the DA system in OCD patients as well as studies investigating cognitive flexibility in OCD.

### DA alterations in OCD

Although there is strong evidence that serotonin plays a role in the treatment of OCD (van Dijk et al., 2010), it is clear that OCD pathophysiology also involves alterations in fronto-striatal circuitry and its neuromodulation by DA. Indirect evidence comes from clinical observations that administration of DA antagonists can improve symptoms in OCD-patients that do not respond to SSRI's alone [(McDougle et al., 2000; Dougherty et al., 2004); see Denys et al. (2004b) for review]. In animals, administration of drugs acting on DAergic receptors and genetic manipulations of DA receptors induces compulsive, stereotypic behaviors similar to the repetitive behaviors of OCD patients (Szechtman et al., 1998; Campbell et al., 1999; Joel and Doljansky, 2003; Denys et al., 2004b; Sesia et al., 2013).

Importantly, direct evidence indicating altered DA signaling in OCD patients is also available. Kim et al. (2003) observed a higher density of the DA transporter (DAT) in the right basal ganglia that normalized after SSRI treatment (Kim et al., 2007). However, these findings were not consistently replicated (Nikolaus et al., 2010): van der Wee et al. (2004) also showed higher binding ratios using OCD patients without co-morbid disorders, but Hesse et al. (2005) observed reduced striatal DAT binding and Pogarell et al. (2003) did not observe differences in DAT availability between OCD patients and healthy controls. The latter authors also reported increased instead of decreased DAT binding after SSRI's.

OCD-patients show reduced binding to D_1_ receptors in caudate nucleus and putamen (Olver et al., 2009) and in anterior cingulate cortex (Olver et al., 2010), although reduced binding does not correlate with symptom severity.

Studies investigating binding to striatal D_2_ receptors in OCD patients present a more consistent picture. The original finding by Denys et al. (2004a) of reduced binding to D_2_ receptors in the caudate nucleus of OCD patients was replicated by others (Perani et al., 2008; Schneier et al., 2008; Denys et al., 2013). In medication-naïve OCD patients, repeated administration of an SSRI increased binding to striatal D_2_ receptors, illustrating that regulation of DA release can be modulated by 5-HT (Moresco et al., 2007).

Taken together, the studies mentioned here described reduced binding to DA receptors in OCD patients, mainly in, but not limited to striatal regions. The most replicated finding is reduced availability of D_2_ receptors in striatal regions. It has been hypothesized that reduced availability of DA receptors in OCD patients could be the result of increased DA release in the striatum (Denys et al., 2004a). However, the observed changes in the DA system do not correlate with symptom severity or duration of illness and it is possible that the DAergic alterations are secondary to diminished serotonergic tone.

### Cognitive flexibility in OCD

Although the repetitive execution of behavioral patterns that is often observed in OCD patients could be defined as inflexible or perseverative behavior, the question is whether this translates to impaired performance on measurements of cognitive flexibility that are currently used in tests of executive functioning.

Findings using the WCST have been contradictory, with some studies observing impaired performance in OCD patients (Lucey et al., 1997; Lacerda et al., 2003; Bohne et al., 2005; Lawrence et al., 2006; Bucci et al., 2007; de Geus et al., 2007; Cavedini et al., 2010), whilst others do not (Gambini et al., 1993; Abbruzzese et al., 1995, 1997; Cavedini et al., 1998; Moritz et al., 2002; Fenger et al., 2005; Henry, 2006). The former studies often describe an increase in the number of perseverative errors. The observation that deficits in flexibility may persist after remission or use of medication and that unaffected family members also show reduced flexibility, suggests that these deficits are trait-like and independent of OCD-symptomatology (Bannon et al., 2006; Cavedini et al., 2010), supporting the hypothesis that inflexible, rigid and habit-like behavior is an endophenotype in OCD.

#### Reversal learning

Alterations in recruitment of fronto-striatal circuitry in the absence of behavioral impairments have been observed in both OCD patients and their unaffected first-degree relatives during reversal learning (Chamberlain et al., 2008). Remijnse et al. (2006) observed attenuated responsiveness of OFC and striatal regions during reward and affective switching in OCD patients with and without comorbidities. In these studies, as well as in others (Valerius et al., 2008; Ersche et al., 2011) no clear evidence for behavioral impairments during task performance was obtained, although OCD patients do show a somewhat slowed response pattern, suggesting they may require more processing time when faced with altered response-reward contingencies. Altered recruitment of fronto-striatal circuitry during these tests suggests that even though overt behavioral performance (i.e., reaction times, number of errors, number of trials required to reach criterion) may not be impaired, the processing of cognitive information is altered in OCD patients during reversal learning.

#### Attentional set-shifting

Performance on tasks that require shifting between different stimulus dimensions does appear to be affected in OCD patients. Behavioral impairments have been observed in OCD patients and unaffected first-degree relatives in an attentional set-shifting task (Veale et al., 1996; Fenger et al., 2005; Watkins et al., 2005; Chamberlain et al., 2006, 2007) but see (Purcell et al., 1998a,b), with some reporting reduced performance on extra-dimensional set-shifts (Veale et al., 1996; Watkins et al., 2005; Chamberlain et al., 2006, 2007) and others on intra-dimensional set-shifts (Veale et al., 1996; Fenger et al., 2005). Response to SSRI-treatment was found to be related to set-shifting ability (Fontenelle et al., 2001).

#### Task switching

Increased switch costs (decreased accuracy or increased response times) have been observed in OCD patients during performance of task switching paradigms (Moritz et al., 2004; Gu et al., 2008; Page et al., 2009). Gu et al. (2008) found an increase in the number of errors made during task-switching trials in OCD patients, but others report slowed responding (Moritz et al., 2004; Remijnse et al., 2013) or no effect (Page et al., 2009). However, when task switching is combined with functional imaging, activity in the dorsal fronto-striatal circuit is consistently found to differ between OCD patients and healthy controls. Whereas activation of the dorsal fronto-striatal circuit is observed in healthy controls during task-switching trials, this is not the case in OCD patients (Gu et al., 2008; Page et al., 2009; Remijnse et al., 2013).

### Summary and conclusion

Several problems arise when interpreting the deficits of OCD patients on cognitive flexibility and the mixed outcomes of the studies investigating these deficits. Next to the influence of medication and the need for careful matching of patient and control groups, the high comorbidity with other psychiatric disorders, in particular depression is an important confounding factor. Although the use of subject groups with OCD as the only clinical diagnosis could be thought of as misrepresentative for the population of OCD patients *because* comorbidity is so common (Olley et al., 2007), the use of well-defined clinical populations in studies combining neuropsychological testing with measurements of brain activity in particular, could contribute to the knowledge about distorted recruitment of frontostriatal circuitry in cognitive flexibility.

As far as we know, studies directly linking measurements of cognitive flexibility to alterations in DA signaling have not been performed in OCD patients. The most consistent alteration in the DA system is changed DA receptor binding, mostly in striatal regions. Replication of these findings, especially of both D_1_ and D_2_ receptor binding, in different OCD samples would enhance our understanding of the contribution of DA to OCD. For performance on cognitive flexibility tasks, behavioral performance on lower order cognitive flexibility (reversal learning) is not altered, whilst OCD patients may be impaired on higher order flexibility tasks (attentional set-shift and task switching). Irrespective of the presence of behavioral impairments, activity and connectivity in neural circuits regulating flexible behavior (OFC-ventral striatum for reversal learning, PFC-dorsal striatum for task-switching) are altered in OCD patients during task execution. Considering the modulatory effect of DA in these neural circuits, it is possible that altered striatal DA contributes to different activity in these circuits during task performance.

## OCD animal models: dopamine and cognitive flexibility

Animal models of psychiatric disorders cannot reflect all aspects of the disease (Nestler and Hyman, 2010). In line with this, OCD models that show a combination of the critical face, predictive and construct validities (Korff and Harvey, 2006; Wang et al., 2009; Fineberg et al., 2011; Albelda and Joel, 2012b) predominantly mirror the compulsive acts of OCD patients. This applies for models based on spontaneous behavior [ethological models, e.g., compulsive dogs, (Vermeire et al., 2012)], behavioral models [e.g., compulsive lever-pressing during signal attenuation in rats (Joel, 2006)], pharmacological models [e.g., quinpirole-induced checking in rats (Szechtman et al., 1998)], and transgenic models [e.g., compulsive grooming in Sapap3-mutant mice, (Welch et al., 2007)]. Compulsive acts are behaviorally and conceptually not always clearly differentiated from simple repetitive behaviors. Repetitive, stereotyped, perseverative, rigid and habitual behavior have been grouped together into (overlapping) clusters of compulsive-like behavior [(Langen et al., 2011; Ting and Feng, 2011; Robbins et al., 2012); for a critical discussion of the distinction between stereotypies and compulsions, see (Lewis et al., 2007)]. These clusters are relevant not only for OCD, but also for other psychiatric disorders and may share a relative DAergic hyperactivity in the basal ganglia (Pitman, 1989). Two recent studies highlight the direct involvement of specific projections from OFC to ventromedial striatum in the regulation of compulsive-like, repetitive behavior in normal mice (Ahmari et al., 2013) and compulsively grooming Sapap-3 mice (Burguiere et al., 2013).

Stereotyped repetitive behavior, in particular, is strongly linked to DA mechanisms (Randrup and Munkvad, 1975; Ridley, 1994). Next to the quinpirole-model (repeated administration of a D_2/3_-selective agonist), the DAT-knockdown mouse that shows stronger and more rigid grooming behavior, has been proposed as an OCD-model based on DA hyperactivation (Berridge et al., 2005). Another model of increased DA-related neuronal activity is the D1CT transgenic mouse, showing repetition of all normal behaviors (Campbell et al., 1999). Most other validated OCD-models also show involvement of DA mechanisms in their compulsive behavior (Joel and Doljansky, 2003; Presti et al., 2003; Albelda and Joel, 2012a; Moreno and Flores, 2012; Vermeire et al., 2012; Sesia et al., 2013), although DA mechanisms were not tested in compulsively grooming transgenic mouse models (Welch et al., 2007; Shmelkov et al., 2010).

The relationship between repetitive behavior and cognitive flexibility as probed in tasks using translationally valid constructs of reversal learning, attentional set-shifting or task switching has received only limited attention. In deer mice, stereotyped jumping was correlated with the number of incorrect responses in a reversal of escape-learning in a water-filled T-maze (Tanimura et al., 2008). BTBR T+ tf.J mice, showing compulsive grooming and increased marble burying, show impaired probabilistic reversal learning (Amodeo et al., 2012). A task probing recurrent perseveration (two-choice task where continuous switching provides the optimal strategy) showed a correlation between stereotyped behavior and recurrent perseveration in farmed minks, but not in ICR CD-1 mice (Gross et al., 2011). Finally, rats compulsively drinking in the schedule-induced polydipsia model displayed increased perseveration during extinction of the 5-choice serial reaction time task and perseveration during extinction of other operant procedures was reported in bank voles (Garner and Mason, 2002) and caged bears (Vickery and Mason, 2005).

However, if we focus on reversal learning, attentional set-shifting or task switching there are no studies available that show task impairments in OCD animal models, let alone impairments related to DA mechanisms. The only possible exception is stereotyped behavior in deer mice, which correlated to the number of incorrect responses during reversal learning and decreased after striatal administration of a D_1_-selective antagonist (Presti et al., 2003; Tanimura et al., 2008), though the relation between reversal learning and DA was not directly investigated.

In conclusion, a possible relation between compulsive behavior and cognitive flexibility, including the possibility that DA mechanisms might play a role in this, did not receive much attention up to now. One can understand that the introduction of translational valid paradigms for cognitive flexibility in exotic species such as bank voles, mink or bears is not an easy task. But using behavioral testing in reversal learning, attentional set-shifting or task switching in rodent OCD-models should be a priority for researchers who want to study the neurobiological underpinnings of OCD.

## Conclusion

Evidence for a role of DA in the control of cognitive flexibility comes from a range of human and animal studies that have been reviewed above. This overview indicates that DA is involved in different facets of cognitive flexibility, including reversal learning, set-shifting and task-switching. Moreover, DA in both cortical and subcortical parts of the corticostriatal circuits seem to be involved in the regulation of these different aspects of cognitive flexibility. The idea that DA facilitates flexibility or switching behavior can be traced back to older studies that used different behavioral paradigms than the studies reviewed here. For example, a role for DA in switching strategies in a swim test was suggested by Cools (1980) and van den Bos and Cools (1989), while the importance of DA in switching (increasing the probability that another behavioral output is chosen) was advocated by Oades (1985).

However, the general picture arises that although DA may facilitate cognitive flexibility, it is not required. Following a variety of manipulations to the DA system the ability to successfully shift behavior following changes in reinforcer contingencies is impaired but not completely absent (in rodents, non-human primates and humans). How does the supportive role of DA in cognitive flexibility (i.e., behavioral adaptation to a change in conditions) compare to its role in initial learning about rewards? The question whether DA is necessary for learning has been addressed by studying acquisition of learning in DA deficient mice—the conclusion was that loss of DA may impair, but does not inhibit reward learning (Berridge, 2005; Robinson et al., 2005; Palmiter, 2008; Darvas and Palmiter, 2010). Animals may become less motivated, but were still able to learn cue-reward associations. Disruption of phasic DA activity by deletion of NMDA-receptors from DA neurons again showed that learning may be retarded, but not inhibited (Zweifel et al., 2009). A recent study using an optogenetics approach showed that phasic DA stimulation may drive associative learning or impair extinction learning, suggesting a causal role for DA (Steinberg et al., 2013). However, DA stimulation could not maintain the original behavior, so that other processes are probably involved as well. During performance of cognitive flexibility tasks, a number of cognitive processes act simultaneously and DA may be especially important to switch behavior rapidly. The contribution of DA to new learning therefore appears to be facilitatory rather than a prerequisite and the supportive role of DA appears to be present both in initial learning and adaptation of learning.

Both pharmacological and genetic studies in human subjects and animals point to a role for D_2_ receptors in the regulation of cognitive flexibility. However, the regulation is not limited to D_2_ receptor activity: D_1_ and D_2_ receptors both contribute and appear to be cooperatively involved in discrimination learning and the flexible adaptation of behavior. One could argue that successful behavioral switching requires three processes that may partly occur in parallel: extinction of the response that is no longer rewarded, behavioral switch to the newly rewarded side and response maintenance. A complication in delineating the contribution of DA to either process is that these processes occur simultaneously during behavioral adaptation. DA signaling through D_1_ receptors may not be essential for switching behavior *per se*, but animal studies suggest that activation of D_1_ receptors contributes to the acquisition and maintenance of a new response, also when acquisition follows a reversal. In contrast, inactivation of D_2_ receptors may allow switching of behavior patterns. The contributions of D_1_ versus D_2_ receptors in the regulation of reward learning and behavior switching has been related to involvement of the direct and indirect pathway of the basal ganglia in these processes, and several models have been put forward to describe the possible components involved in regulating this behavior (Frank and Claus, 2006; Hong and Hikosaka, 2011). In general these models assume the presence of D_1_ receptors in the direct pathway (direct projections from striatal medium spiny neurons (MSN) to the substantia nigra) and expression of D_2_ receptors on MSN's of the indirect pathway (projections from MSN to substantia nigra via the globus pallidus) (Deng et al., 2006). Because binding affinity differs for D_1_ and D_2_ receptors (Richfield et al., 1989), fluctuations in DA levels during different stages of discrimination and reversal learning may result in different activation of D_1_ (direct pathway) or D_2_ (indirect pathway) expressing neurons. When a reward is presented unexpectedly, or when a stimulus that predicts reward is presented, a transient increase in DA release occupies low affinity D_1_ receptors and activates the direct pathway, allowing facilitation of response execution and prompting reward-related learning. Switching of behavioral patterns on the other hand might require reduced occupancy of high affinity D_2_ receptors. Omission of an expected reward following altered reinforcer contingencies results in transient reductions in striatal DA levels and diminished inhibition of the indirect pathway by D_2_ receptors, resulting in inhibition of the previously successful response. Both facilitation of behavioral adaptation by deactivation of striatal D_2_ receptors and facilitation of the acquisition of the “new” behavioral response by striatal D_1_ activation suggests the importance of phasic fluctuations in striatal DA levels during execution of cognitive flexibility. This may be illustrated for the D_2_-mediated response: both continuously higher and lower tonic D_2_ activation could impair detection of the transient reduction of DA. As tonic DA may be related to general synaptic factors such as synthesis capacity, uptake activity and metabolic efficiency, all these factors may influence flexible responding through D_2_ receptor dependent transmission. However, it is difficult to separate tonic from phasic DA signaling with most manipulations used. Tonic prefrontal DA (Seamans and Yang, 2004) probably contributes as well. In addition, activation of D_1_/D_2_ receptors in prefrontal regions may differ from the activation in striatal regions. It has been suggested, for example, that D_2_ stimulation in prefrontal regions may facilitate flexible behavior (Durstewitz and Seamans, 2008) whereas in striatal regions, deactivation of D_2_ receptors is suggested to facilitate cognitive flexibility (Yawata et al., 2012). The combined study of genetic effects on behavioral performance and patterns of neural activation also suggests that although DA genotype may primarily affect expression of DA related genes in either striatal or prefrontal areas, functional effects of DA genotype are not limited to either region but are observed throughout the frontostriatal circuit. Genetic and imaging studies suggest that DA in ventral regions of the striatum (or connections between PFC and ventral striatum) contributes to reversal learning (lower order complexity), whereas DA in dorsal regions may be more important for attentional set-shifting and task switching (higher order complexity). However, animal studies have also described effects of DA in the NAC on attentional set-shifts and animals that only have DA signaling in dorsal striatal regions are able to learn a reversal. In addition, in human imaging studies it is not always clear if activation is limited to either ventral or dorsal striatum because analysis was limited to that particular striatal region or because the other striatal region was not activated. Therefore, it appears to be more likely that the relative activation of D_1_/D_2_ in prefrontal and striatal regions as well as the interaction with other neuromodulators (5-HT, NA) determines the control of cognitive flexibility. Considering the complexity of DA modulation in frontostriatal circuitry (Seamans and Yang, 2004), it may not be surprising DA modulation in neither frontal nor striatal regions that exclusively determines behavioral performance on tasks of cognitive flexibility.

So how do these findings relate to altered cognitive flexibility in OCD patients? If cognitive flexibility can indeed be used as an endophenotype for OCD, do the alterations in DA signaling that have been observed in OCD patients comply with the proposed role for DA in cognitive flexibility? The most replicated alteration in the DA system of OCD patients is reduced binding to D_2_ receptors in the striatum. A questions remains, how reduced D_2_ receptor binding relates to DAergic activity *in vivo*. A reduction in binding potential to D_2_ receptors may result from increased striatal DA levels or altered availability of D_2_ receptors. In both cases, reduced flexibility could be expected. However, behavioral performance (i.e., accuracy) on reversal learning tasks is not impaired in OCD patients. On reversal learning tasks, if any behavioral effect is found, it is a slowing of response times rather than an effect on the amount of errors that are made. Differences in accuracy have been observed in attentional set-shifting and task switching paradigms. It is possible that reversal learning may be a paradigm that is too simple for gross behavioral abnormalities to be observed in OCD patients. Increased reactions times on flexibility tasks, however, do suggest altered cognitive processing in OCD patients during cognitive flexibility and the measurement of reaction times should therefore be included in studies investigating differences in cognitive flexibility between healthy controls and OCD patients. The altered recruitment of frontostriatal circuitry during the execution of reversal learning as well as task switching is another indication for altered cognitive processing in OCD patients. Altered DA signaling is a potential contributor to changes in frontostriatal activity when performing cognitive tasks. Altered activity in the frontostriatal circuit (OFC-ventral striatum) during reversal learning, as observed in OCD patients is also found in subjects with polymorphisms in the D_2_ gene that result in reduced binding to D_2_ receptors. Most likely, however, abnormalities in prefrontal regions and 5-HT modulation in OCD patients also contribute.

An important step in investigating the possibility of altered cognitive processing in cognitive flexibility tasks as an endophenotype for OCD would be the replication of studies using cognitive flexibility tasks in OCD patients with the use of strictly defined patient and control groups. Considering that altered neural correlates of OCD could be symptom dimension-specific (van den Heuvel et al., 2009), separate study of the different symptom dimensions contributes to the identification of possible endophenotypes. Preferably, these studies combine behavioral testing with measurements of brain activity and/or DA activity to further investigate the neurobiological basis of altered cognitive processing during cognitive flexibility tests in OCD patients.

### Conflict of interest statement

The authors declare that the research was conducted in the absence of any commercial or financial relationships that could be construed as a potential conflict of interest.
